# Identification of Hub Genes and Pathways in Preinfusion Chimeric Antigen Receptor (CAR) T-cell Products Associated With Cytokine Release Syndrome

**DOI:** 10.7759/cureus.82155

**Published:** 2025-04-12

**Authors:** Somia A Khalafallah, Anas Mohammed Elzein, Israa M Faris, Gamila A Attaelmanan, Marwa F. Alamin, Alaa Almleeh, Mohammed Bader, Mohamed Alfaki

**Affiliations:** 1 Department of Hematology and Immunohematology, Ibn Sina University, Khartoum, SDN; 2 Department of Molecular and Cellular Medicine, Institute of Translational Medicine, Zhejiang University School of Medicine, Hangzhou, CHN; 3 Department of Fertilization and Artificial Insemination, Istanbul University-Cerrahpasa, Istanbul, TUR; 4 Department of Hematology, Al Neelain University, Khartoum, SDN; 5 Department of Molecular Biology, Institute of Endemic Diseases, University of Khartoum, Khartoum, SDN; 6 Department of Biotechnology, School of Pharmacy, Ahfad University for Women, Omdurman, SDN; 7 Department of Surgery, Faculty of Medicine, University of Khartoum, Khartoum, SDN; 8 Department of Software Engineering, Faculty of Computer Science, Al Neelain University, Khartoum, SDN

**Keywords:** bioinformatics analysis, car t-cell therapy, cytokine release syndrome, enrichment pathways analysis, hub genes

## Abstract

Background: Chimeric antigen receptor (CAR) T-cell therapy has transformed cancer management over the past decades, offering new hope to many patients. However, its effectiveness is often limited due to cytokine release syndrome (CRS), a life-threatening inflammatory response. Despite its clinical relevance, the molecular mechanisms underlying CRS, specifically in CAR T-cell products, remain poorly understood. This study aims to identify hub genes and pathways in preinfusion CAR T-cell products associated with CRS development and evaluate their potential as therapeutic targets through drug-gene interaction analysis and immune cell correlation profiling.

Methods: We examined gene expression data from 43 preinfusion clusters of differentiation 22 of CAR T-cell samples (CD22+), sourced from the Gene Expression Omnibus dataset GSE200296. Using the linear models for microarray data package, we identified differences in gene expression and conducted enrichment analyses to explore relevant biological pathways, including Kyoto Encyclopedia of Genes and Genomes and Gene Ontology terms. We built protein-protein interaction networks using the Search Tool for Retrieval of Interacting Genes/Proteins database to understand how these genes interact and pinpointed central "hub genes" with Cytoscape and the cytoHubba plugin. Our findings were validated using the GeneCards database (Weizmann Institute of Science, Israel) and an independent CRS-related dataset (GSE164805). Additionally, we analyzed immune cell populations and explored potential drug-gene interactions.

Results: Our study identified 24 genes with changed expression levels: 16 were downregulated and eight were upregulated. We identified five hub genes, interleukin (IL)1B, IL15, CD276, NCR2, and CCL17, as key contributors in CRS, which were primarily implicated in immune-related pathways, including cytokine-cytokine receptor interactions, IL17 signaling, and TNF signaling. These genes were especially expressed in monocytes, macrophages, and dendritic cells, confirming that those immune cell types play a critical role in CRS development. Through drug-gene interaction analysis, we found prospective therapies, such as enoblituzumab (targeting CD276) and canakinumab (targeting IL1B), which might assist in reducing CRS severity.

Conclusion: The study highlights IL1B, IL15, CD276, NCR2, and CCL17 as key CRS genes in preinfusion CAR T-cell products. Their dysregulation activity may contribute to the increased inflammation noted in CRS, pointing to a loss of regulatory control. Bringing us closer to better patient outcomes, these findings not only suggest that these genes could serve as valuable biomarkers for predicting CRS but also open the way for the development of more precise treatments such as combining drugs such as enoblituzumab and canakinumab, which might assist in reducing CRS severity and making CAR T-cell therapy safer and more effective, ultimately improving patient lives.

## Introduction

Chimeric antigen receptor (CAR) T-cell therapy is a significant revolution in cancer immunotherapy, giving strength to patients with refractory or relapsed hematologic malignancies. CAR T-cell therapy has demonstrated exceptional efficacy in treating cancer, including B-cell acute lymphoblastic leukemia and non-Hodgkin lymphoma, by genetically modifying T cells that show synthetic receptors that precisely target tumor antigens [1,2]. Despite its transformative avenues, CAR T-cell therapy is typically associated with substantial toxicities, with cytokine release syndrome (CRS) the most notable [3]. CRS represents a systemic inflammatory response triggered by the rapid activation and proliferation of CAR T-cells, culminating in the abundant release of proinflammatory cytokines comprising interleukin (IL)6, IL1, interferon-gamma, and other cytokines, along with macrophage (MF) activation, causing extensive inflammation [4]. This phenomenon, generally called a cytokine storm, shows a variety of clinical symptoms ranging from mild flu-like signs to critical, life-threatening problems such as hypotension, breathing distress, and multiorgan dysfunction [5]. Although the use of IL6 receptor antagonists, such as tocilizumab, has reduced the side effects of CRS, the fundamental molecular mechanisms and genetic factors that affect patient-specific therapeutic responses and disease severity remain poorly understood [6]. The resulting sign suggests that the genetic and molecular profiles of preinfusion CAR T-cell products could have a crucial role in evaluating the risk and severity of CRS [7]. For instance, the expression levels of certain cytokines, chemokines, and immune checkpoint molecules in preinfusion CAR T-cells have been related to the subsequent development of CRS [8,9]. However, there is a lack of understanding of the key genes and pathways associated with CRS pathogenesis, particularly in the circumstances of preinfusion CAR T-cell products [9]. Identifying these molecular pathways could provide valuable insights into the fundamental mechanisms of CRS and offer potential therapeutic targets to mitigate its severity. Our research aimed to uncover key genes and molecular pathways within preinfusion CAR T-cell products that correlate with the development of CRS and evaluate their potential as therapeutic targets through drug-gene interaction analysis and immune cell correlation profiling. A combination of bioinformatics approaches, including differential gene expression analysis, pathway enrichment, and protein-protein interaction networks, was employed to elucidate the genetic landscape of CRS-associated genes. Furthermore, we validated our findings using independent datasets and examined the potential genes as therapeutic targets. Our results provide new insights into the molecular mechanisms that handle CRS and offer a foundation for developing strategies to increase the safety and efficacy of CAR T-cell therapy.

## Materials and methods

Identification of DEG analysis

On September 10, 2024, the CRS-related dataset GSE200296 was retrieved using the Gene Expression Omnibus (GEO) database (https://www.ncbi.nlm.nih.gov/geo/), which includes 43 CD22+ CAR-T cell preinfusion products. Each specimen is correlated with specific CRS classifications (without CRS, CRS grade 1, CRS grade 2, CRS grade 3, and CRS grade 4). We established the group without CRS as a control cohort comprising three specimens, while the remaining classifications constitute the CRS case-cohort, totaling 40 specimens. The data from the dataset were analyzed using the linear models for microarray data (limma) (version 3.60.6) in the R package (R Foundation for Statistical Computing, Vienna, Austria) [10] and in Bioconductor [11]; the differential expression genes (DEGs) were recognized by applying Benjamini-Hochberg correction to control the false discovery rate, with a threshold of log_2_ fold change |log_2_ (FC)| > 0.585. A p value of <0.05 was considered statistically significant [12]. The DEGs volcano plot was created using EnhancedVolcano (version 1.22.0; GitHub, San Francisco, CA).

Enrichment analysis

To identify the biological and molecular processes linked to DEGs, we conducted enrichment analysis using the Kyoto Encyclopedia of Genes and Genomes (KEGG) pathways. Additionally, the DEGs underwent functional enrichment analysis to determine significantly enriched Gene Ontology (GO) terms, encompassing biological process, molecular function, and cellular component categories. The analysis utilized the Enricher online database (https://maayanlab.cloud/Enrichr/), with p values <0.05 considered significant, enrichment outcomes exported as Excel files (Microsoft Corporation, Redmond, WA) for visual representation [13]. Visual depictions were created using the ggplot2 package (version 3.5.1; R Foundation for Statistical Computing, Vienna, Austria) and SRplot (https://www.bioinformatics.com.cn/srplot) [14,15].

Protein-protein interaction

The Search Tool for Retrieval of Interacting Genes/Proteins (STRING; version 12.0) database, available at https://string-db.org/, contains both known and predicted protein-protein interaction (PPI) [16]. It was employed for PPI network construction, then the generated networks were imported into Cytoscape (version 3.10.2; Institute for Systems Biology, Seattle, WA) for further analysis and visualization [17]. Hub genes were recognized using the cytoHubba plugin in Cytoscape by applying multiple algorithms such as degree, maximal clique centrality (MCC), and betweenness centrality [18,19].

Validation of hub genes

Hub genes were validated by cross-referencing with the GeneCards database (version 5.21; Weizmann Institute of Science, Israel) (https://www.genecards.org/) to assess their relevance in CRS. Further validation was carried out using the GSE164805 dataset, which contains data related to CRS in COVID-19 [18]. Therefore, it could serve as a valuable resource for validating hub genes associated with CRS.

The immune cell population

The hub gene expression data for immune cell populations were retrieved from the Immunological Genome Project (ImmGen) data browsers (https://www.immgen.org/) by selecting numerous kinds of data to browse microarray version 2 (Illumina, San Diego, CA) data, which involves expression profiles of sorted immune cell types, such as natural killer (NK) cells, dendritic cells (DCs), MFs, monocytes (Mo), B cells (BA), and granulocytes (GN) [20].

The drug-gene interaction

We utilized the Drug-Gene Interaction Database (DGIdb) associated with drugs to explore potential therapeutic targets for the validated hub genes, ranking them by interaction score to prioritize the most promising candidates [21]. To better understand the relationships, we visualized the overlapping drugs and potential targets using Cytoscape, creating a clear and interactive network highlighting key therapeutic opportunities.

Statistical analysis

The DEGS were identified using the limma package, which applies the empirical Bayes moderated t-test to compute p values and log₂ fold changes. Genes with p < 0.05 and |log₂ FC| > 0.585 were considered significant. Subsequent pathway and GO enrichment analyses were performed using Fisher’s exact test (p < 0.05). A PPI network was then constructed using the STRING database with a confidence score threshold of >0.4. The immune cell population analysis using the one-way analysis of variance to compare the immune cells (p < 0.05) is considered significant.

## Results

Identification of DEGs

**Figure 1:**
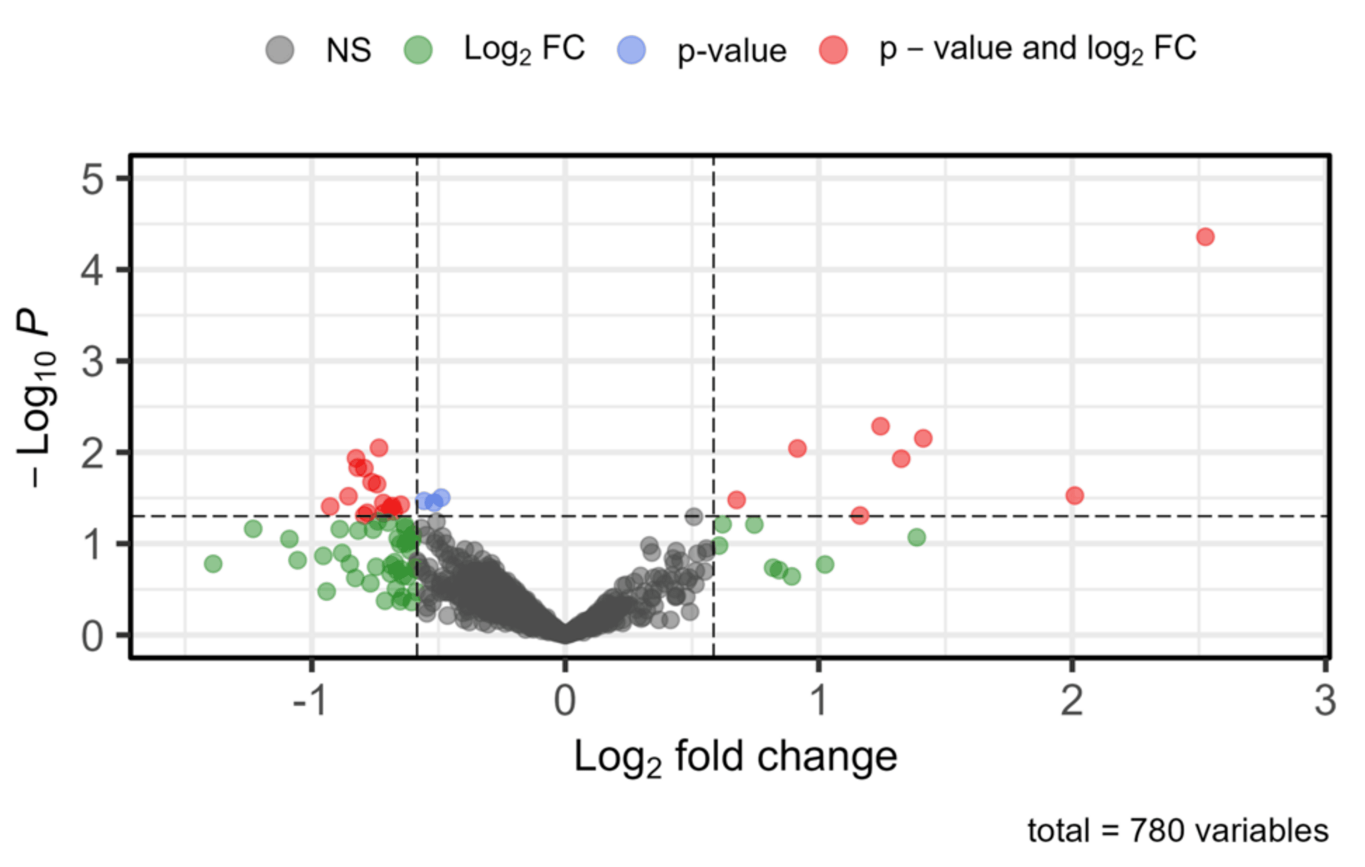
Enhanced volcano plot of DEGs from the GSE200296 dataset Genes are classified based on specific thresholds (p < 0.05, |log_2_ FC| > 0.585). Red dots indicate genes significant by both p value and log_2_ FC, blue dots represent genes significant only by p value, green dots show genes significant only by log_2_ FC, and gray dots represent nonsignificant genes DEG: differential expression gene; log_2_ FC: log_2_ fold change; NS: not significant

Comprehensive gene expression data were extracted from the selected ID GSE200296 from the GEO database. Differential expression analysis was conducted using a cutoff p value <0.05 and |log_2 _FC| > 0.585. This analysis identified 24 DEGs, consisting of 16 downregulated genes and eight upregulated genes, while 756 genes did not meet the significance criteria. These findings are exhibited in Figure 1 and detailed in Tables 1, 2.

**Table 1:** DEGs for downregulated genes LogFC: log fold change; DEGs: differential expression genes

Gene symbol	LogFC	p value
CD276	-0.73508	0.008939
CCL17	-0.82587	0.011616
GLS2	-0.81917	0.014704
CD300C	-0.79288	0.014925
RARB	-0.76423	0.021187
CLDN3	-0.74297	0.022349
SLC7A5	-0.85558	0.030304
NCR2	-0.71805	0.035876
IL15	-0.64907	0.037522
IL36RN	-0.68389	0.03875
TNFRSF18	-0.92778	0.039183
LNX1	-0.68905	0.041056
CCL21	-0.71677	0.04651
IL1F10	-0.79344	0.049159
IL1B	-0.67703	0.044315
CMKLR1	-0.78152	0.04589

**Table 2:** DEGs for upregulated genes LogFC: log fold change; DEGs: differential expression genes

Gene symbol	LogFC	p value
PFKFB4	2.525555	0.0000438
ZBTB16	1.243859	0.005185
TRGC1	1.412222	0.007032
TRAV26-1	0.915969	0.009074
FOSB	1.325354	0.011757
HLA-DQA1	2.010159	0.029765
PDK1	0.675913	0.033218
TRDV1	1.162902	0.049205

KEGG and GO analysis for downregulated genes

**Figure 2:**
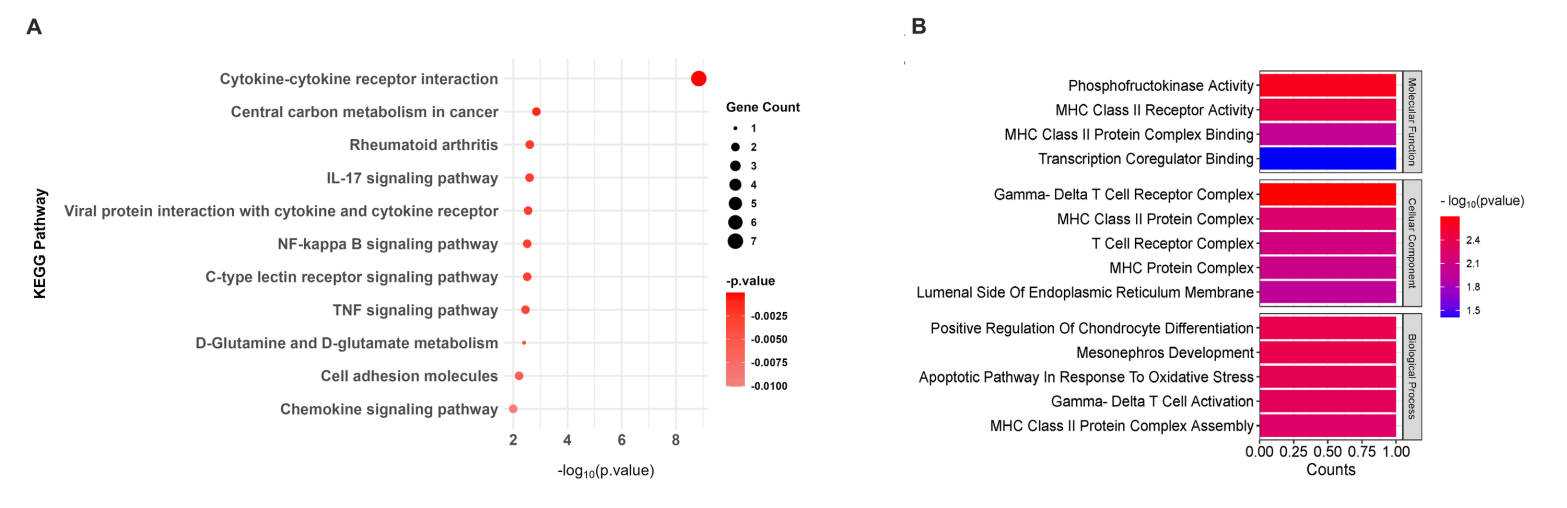
Enrichment analysis for downregulated genes. (A) The KEGG pathways for downregulated genes (red dot plot), with dot size representing the number of genes associated with each pathway and color intensity indicating the statistical significance (p value). Pathways with higher gene counts and greater significance are shown as larger and darker dots. (B) GO terms associated with downregulated genes. The bar graphs represent the counts of genes associated with each GO term, and the color gradient from red to blue indicates the significance level of the p value, with red representing the most significant terms and blue representing less significant terms KEGG: Kyoto Encyclopedia of Genes and Genomes; IL: interleukin; NF kappa B: nuclear factor kappa B; TNF: tumor necrosis factor; MHC: major histocompatibility complex

KEGG pathway analysis of downregulated genes revealed influential enrichment in immune-related pathways, with the cytokine-cytokine receptor interaction pathway being the most prominent (p = 3.11 × 10^-05^), including key genes like IL15, IL1B, and CCL17, which are crucial for immune signaling. Additionally, the IL17 signaling pathway (p = 0.00022) and tumor necrosis factor (TNF) signaling pathway (p = 0.00031) were also significantly enriched, emphasizing their roles in inflammatory processes (Figure 2A). GO analysis further supported these findings by highlighting their functional roles through three main categories. The most significant term in the molecular function category was cytokine activity (p = 2.13 × 10^-07^), involving key genes such as IL15, IL1B, and CCL17, which play essential roles in cytokine signaling and immune regulation. The top enriched term for the biological process category was cytokine-mediated signaling pathway (p = 1.31 × 10^-06^), emphasizing the involvement of genes like IL1B and CD276 in modulating immune responses and inflammatory processes. In the cellular component category, the most notable term was the external side of the apical plasma membrane (p = 0.00399), indicating the role of downregulated genes in cell surface interactions and membrane-related functions. Together, these findings underscore the critical involvement of downregulated genes in immune regulation, inflammation, and cellular communication (Figure 2B).

KEGG and GO analysis for upregulated genes

**Figure 3:**
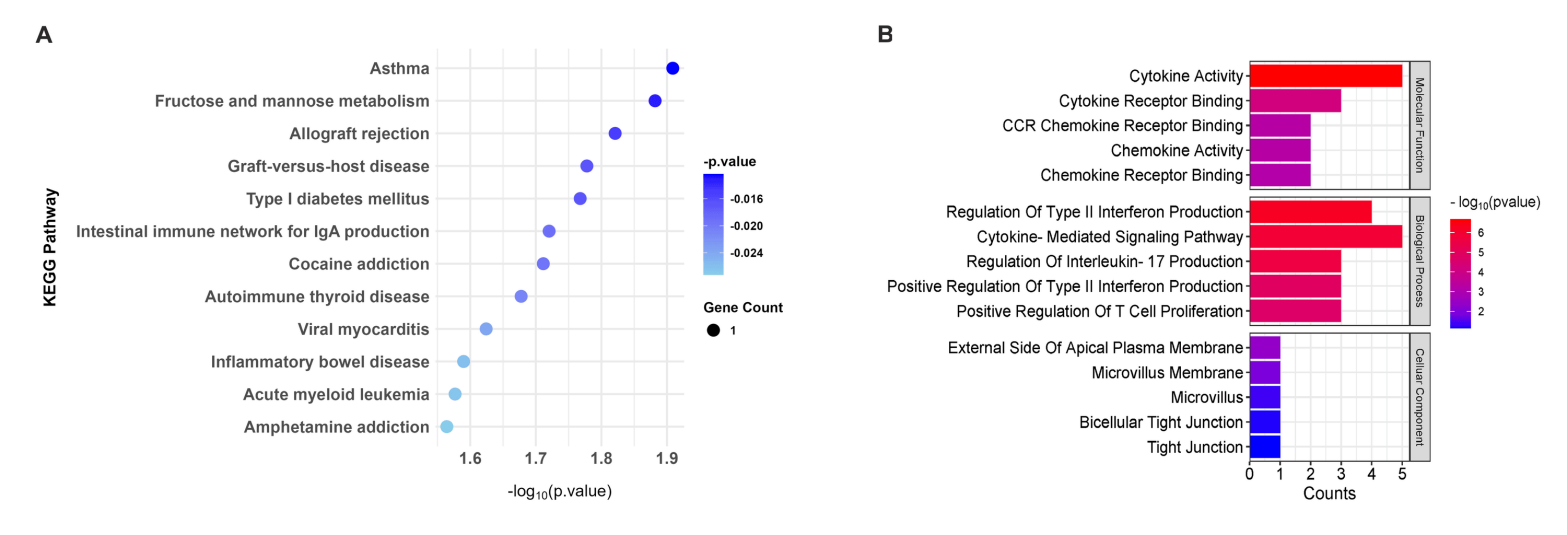
Enrichment analysis for upregulated genes. (A) The KEGG pathways for upregulated genes (blue dot plot), with dot size representing the number of genes associated with each pathway and color intensity indicating the statistical significance (p value). Pathways with higher gene counts and greater significance are shown as larger and darker dots. (B) GO terms associated with upregulated genes. The bar graphs represent the counts of genes associated with each GO term, and the color gradient from red to blue indicates the significance level of the p value, with red representing the most significant terms and blue representing less significant terms KEGG: Kyoto Encyclopedia of Genes and Genomes; GO: Gene Ontology; IgA: immunoglobulin A; CCR: chemokine receptor

KEGG pathway analysis of upregulated genes highlighted their roles in metabolic and immune-related pathways, including fructose and mannose metabolism (p = 0.0131), immune pathways like allograft rejection (p = 0.0151), and type I diabetes mellitus (p = 0.0171) (Figure 3A). GO analysis revealed their involvement in sugar-phosphatase activity (p = 0.0024) and MHC class II receptor activity (p = 0.0036), linking them to metabolic and immune processes. Additionally, upregulated genes were associated with gamma-delta T cell activation (p = 0.0048) and MHC class II protein complex assembly (p = 0.0056), emphasizing their roles in immune regulation and antigen presentation. These findings suggest that upregulated genes contribute to metabolic reprogramming and immune-mediated disease mechanisms (Figure 3B).

The PPI and identification of hub genes

The STRING database (version 12.0) was employed to elucidate the PPIs among the identified DEGs with a confidence score threshold set at 0.4. This analytical approach yielded a PPI network comprising 22 nodes and 33 edges, which encapsulates the interactions between the proteins encoded by the DEGs. The network was rendered visually utilizing Cytoscape software, providing a detailed representation of the connectivity and interactions among the proteins.

**Figure 4:**
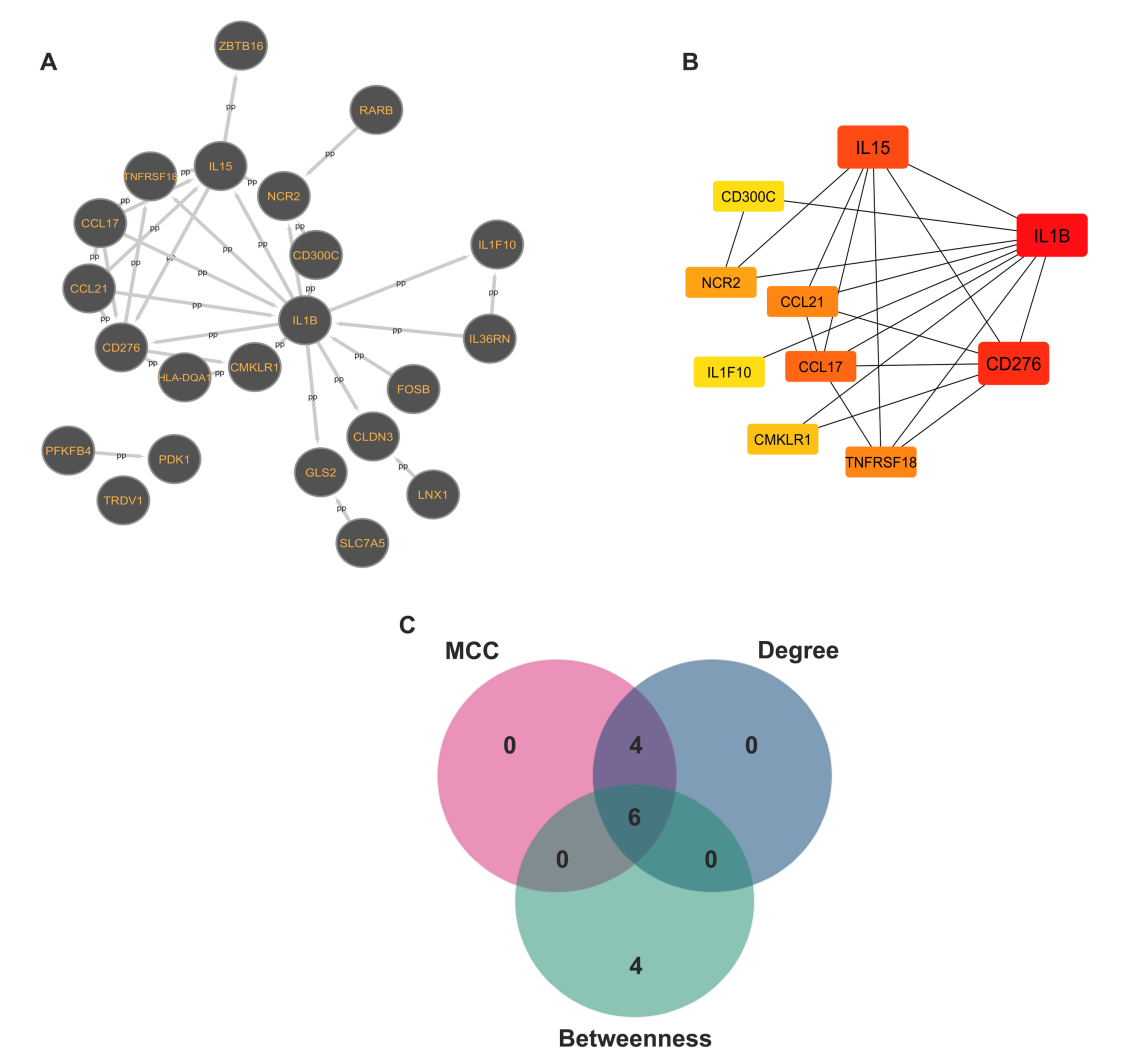
The PPI network and identification of feature genes. (A) The PPI network was constructed using the STRING database. (B) The top 10 feature genes were ranked based on their centrality using the MCC algorithm in the cytoHubba plugin shortest path method. Node colors range from deep red (higher rank) to lighter shades, highlighting their relative importance within the network. (C) Overlap Venn diagram of the top-ranked genes identified using the Degree, MCC, and Betweenness centrality algorithms in cytoHubba PPI: protein-protein interaction; MCC: maximal clique centrality

Through the application of the cytoHubba plugins, which include algorithms such as MCC, Degree, and Betweenness centrality, six prevalent hub genes were shared: IL1B, IL15, CD276, NCR2, CCL17, and CMKLR1 (Figure 4C). Notably, IL1B emerged as the foremost hub gene across all analytical algorithms, emphasizing its pivotal role within the network. Additionally, IL15 and CD276 also attained high rankings, suggesting their importance as principal regulators. Genes such as CCL17 and NCR2 exhibited moderate significance (Table 3). These observations accentuate IL1B, IL15, and CD276 as essential contributors to CRS during CAR T-cell therapy, positioning them as promising candidates for subsequent research and therapeutic advancement.

**Table 3:** CytoHubba plugin shared hub genes MCC: maximal clique centrality; N/A: not available

Gene symbol	MCC score	Degree score	Betweenness score
IL1B	59	13	224.17
IL15	51	7	43.83
CD276	52	7	27.17
NCR2	5	4	36
CCL17	48	5	0.5
TNFRSF18	24	4	N/A
CCL21	24	4	N/A
CMKLR1	4	3	10.33

Validation of hub genes

**Figure 5:**
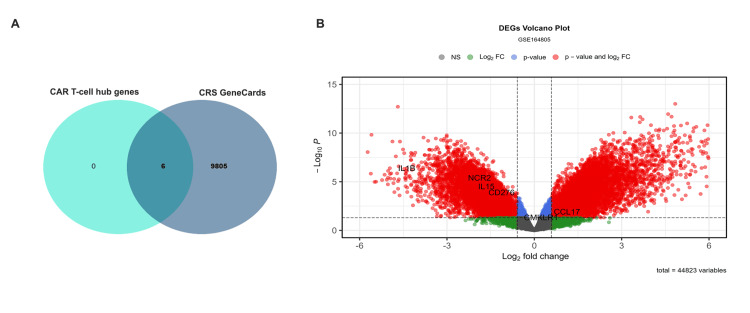
Validation of CRS hub genes. (A) Overlap between the six hub genes identified in CAR T-cell therapy and 9,810 CRS-associated genes from the GeneCards database, confirming their relevance to CRS pathogenesis. (B) Volcano plot of DEGs from the GSE164805 dataset, highlighting the expression patterns of the six hub genes (p < 0.05, |log2FC| > 0.585). Red dots indicate genes significant by both p value and log2 FC, blue represents genes significant only by p value, green indicates genes significant only by log2 FC, and gray represents nonsignificant genes CAR: chimeric antigen receptor; CRS: cytokine release syndrome; DEGs: differential expression gene; log_2_ FC: log fold change; NS: not significant

The six hub gene validation was done by cross-referencing them with 9,810 genes associated with CRS, which was retrieved from the GeneCards database using the search key term "cytokine release syndrome" in September 2024. This analysis confirmed that all six hub genes are linked to CRS (Figure 5A). For further validation, we utilized an independent dataset, the GSE164805, which is derived from COVID-19 patients and shares pathophysiological similarities with CRS [22]. Among these six hub genes, five (IL1B, IL15, CD276, NCR2, and CCL17) exhibited significant differential expression (p < 0.05, log_2 _FC > 0.585). Interestingly, CCL17 showed contrasting regulation patterns between the two datasets: it was downregulated in our dataset (GSE200296) but upregulated in the GSE164805 dataset. This discrepancy may reflect context-specific regulatory mechanisms or differences in the underlying pathophysiology of the studied conditions [22]. These findings highlight the complex role of CCL17 in immune dysregulation and suggest that disease-specific factors may influence its expression (Figure 5B). These findings reinforce the relevance of these hub genes in CRS pathogenesis. Subsequent downstream analyses were conducted to elucidate the precise molecular mechanisms through which these genes contribute to CRS development in the context of CAR T-cell therapy.

Enrichment analysis for five hub genes

**Figure 6:**
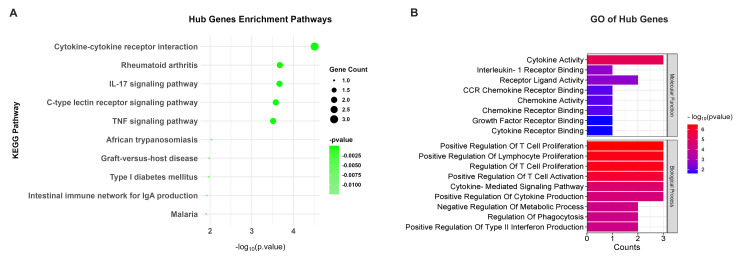
Functional enrichment analysis of hub genes. (A) Enriched pathways associated with the hub genes, including cytokine-cytokine receptor interaction, rheumatoid arthritis, and IL17 signaling pathway. (B) GO analysis of hub genes, highlighting molecular functions and biological processes. Bar graphs represent gene counts and -log10 (p value) for significant terms IL17: interleukin 17; TNF: tumor necrosis factor; GO: Gene Ontology; CCR: chemokine receptor

The five hub genes, IL1B, IL15, CCL17, CD276, and NCR2, were found significantly involved in pathways and activities that are essential to the pathophysiology of CRS using KEGG enrichment analysis. IL1B, IL15, and CCL17 are mainly associated with the cytokine-cytokine receptor interaction pathway (p = 3.11 × 10^-05^). These genes were also implicated in inflammatory pathways such as the IL17 signaling pathway (p = 0.00022) and TNF signaling pathway (p = 0.00031), which are known to drive excessive cytokine production and immune dysregulation, hallmarks of CRS [23] (Figure 6A). GO analysis further demonstrated their functional processes and, with notable enrichment in cytokine activity (p = 6.84 × 10^-06^) and positive regulation of T cell proliferation (p = 3.26 × 10^-07^), underscored their contribution to immune cell activation and cytokine storm development. These findings suggest that the hub genes regulate cytokine signaling and immune cell proliferation, driving the hyperinflammatory state observed in CRS. They highlight their potential as therapeutic targets to mitigate CRS severity in CAR T-cell therapy.

The immune cell population of hub genes

**Figure 7:**
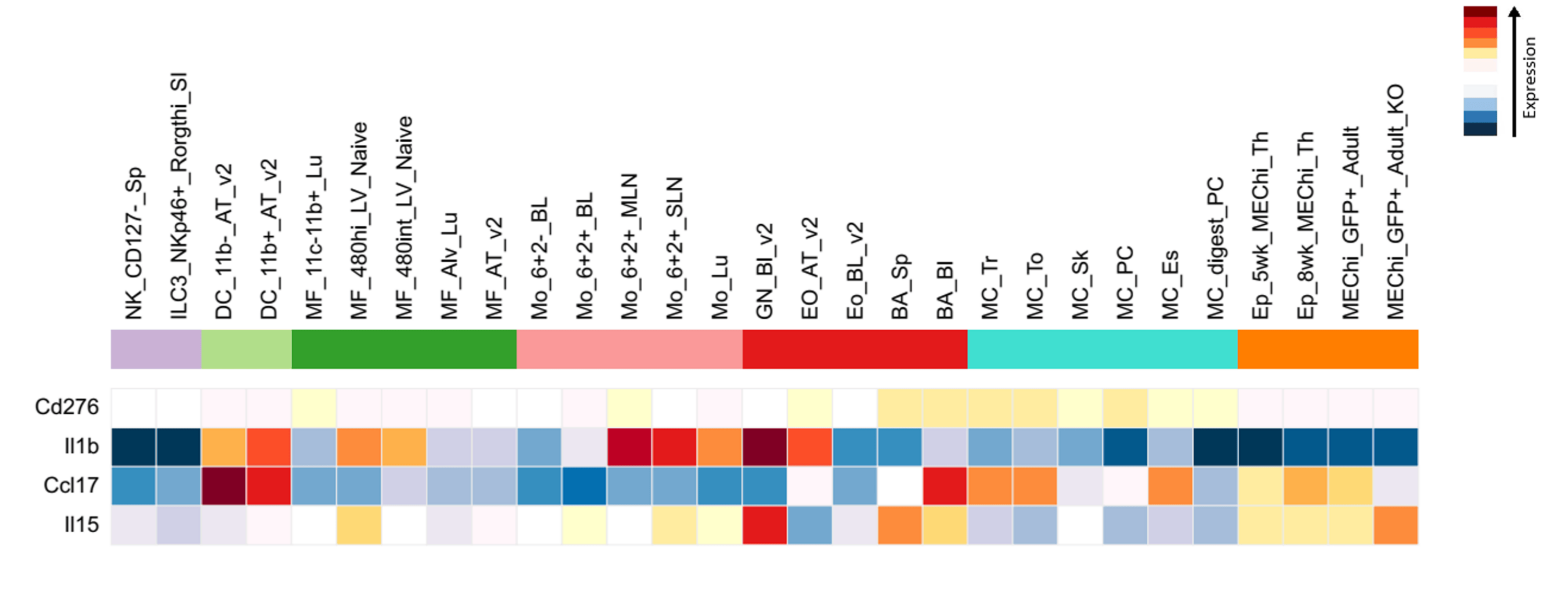
Heatmap of hub gene expression across immune cell populations The heatmap shows the expression levels of hub genes across different immune cell types. Expression levels are color-coded, with deep red indicating high gene-level expression and deep blue indicating low-level expression. The Y-axis represents the hub genes, while the X-axis represents the immune cell populations NK: natural killer; ILC: innate lymphoid cells; DC: dendritic cells; MF: macrophage; Mo: monocytes; BA: B cells; GN: granulocytes; Eo: eosinophils; MC: mast cells; Ep: erythroid progenitors

By using the ImmGen database, we examined the expression of these hub genes in various immune cell types to gain a better understanding of how they can contribute to CRS. Interestingly, we found elevated expression levels of IL1B and CCL17 in all Mo, MFs, GN, and DCs, indicating that these cells may be key players in the inflammatory storm that occurs in CRS. Conversely, IL15 was substantially expressed in T-cells and DCs, highlighting its function in immune cell activation that may also enhance CRS. In the meantime, DCs and MFs also exhibited high levels of CD276, a protein that aids in controlling immunological responses, which may have served as a check on the immune system during CRS. Interestingly, these genes were barely active in NK cells, BA, and GN, which seem less involved in the cytokine chaos; notably, no data were found for NCR2 (Figure 7). Together, these findings point to Mo, MFs, and DCs as key players in CRS, offering clues for how we might target these cells to reduce CRS severity in CAR T-cell therapy.

The drug-gene interaction

**Figure 8:**
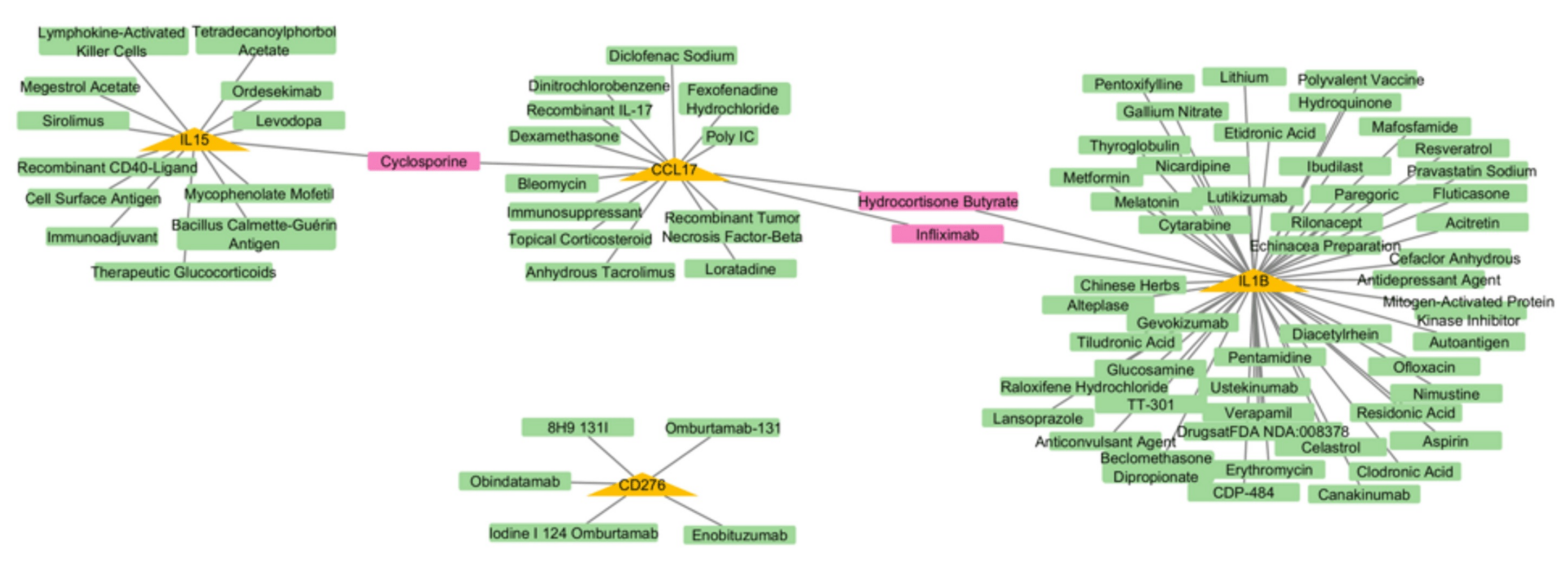
The drug-gene interaction network of hub genes The network illustrates interactions between hub genes highlighted in yellow triangles and potential therapeutic drugs shown in green rectangles. Shared drugs that interact with multiple hub genes are highlighted in pink rectangles

The drug-gene interaction analysis identified 85 medications associated with four of the five hub genes (IL1B, IL15, CCL17, and CD276) using the DGIdb. The highest interaction score was observed for enoblituzumab, an inhibitor of CD276, with a score of 31.32, suggesting it is a potent therapeutic candidate for targeting this immune checkpoint molecule. Other notable interactions included canakinumab (IL1B inhibitor, score: 2.55) and ordesekimab (IL15 inhibitor, score: 3.72), both of which are known to modulate inflammatory responses and may have potential applications in mitigating CRS. Interestingly, CCL17 showed interactions with drugs such as recombinant TNF-beta (score: 3.48) and cyclosporine (score: 0.08), which are implicated in immune regulation (Figure 8). However, no drug interactions were identified for NCR2, highlighting a gap in current therapeutic options for this gene. Most identified drugs functioned as inhibitors, aligning to suppress overactive immune responses during CRS. These findings provide a comprehensive map of potential therapeutic agents targeting hub genes involved in CRS, offering a foundation for further experimental validation and drug remodeling strategies.

## Discussion

Recent advancements in cancer therapeutics have marked a pivotal change, particularly due to the rise of CAR T-cell therapy, which delivers lasting clinical results for specific cancers. Nevertheless, this innovative therapeutic approach is frequently associated with considerable toxicities, among which CRS is the most prevalent [4]. CRS is a systemic inflammatory response characterized by excessive cytokine production and immune cell activation, which, while potentially beneficial in combating cancer, can escalate to life-threatening levels if uncontrolled [6]. One of the key steps toward understanding the mechanisms of CRS-associated CAR-T cell therapy is to explore the driver genes that influence CRS and increase its severity. However, the primary mechanisms that augment the CRS after the CD22+ CAR-T cell therapy are not well known. Therefore, further genetic studies could provide insights into how to explore CRS more effectively. This study revealed that five hub genes, IL1B, IL15, CD276, NCR2, and CCL17, were identified using the GSE200296 dataset and were enriched in pathways related to immune responses and inflammation. Interestingly, the dataset in this study is described as preinfusion CAR-T cell samples. In this context, our findings reflect the prime genes that could be potential candidates for addressing CRS associated with CAR T-cell therapy. Our analysis discovered that hub genes were downregulated in preinfusion CAR T-cell samples, and dysregulation confirms the complexity of CRS pathogenesis. Therefore, this downregulation could be a reaction of engineering strategy to modulate immune response and loss of regulatory control, which triggers potential compensatory inflammatory mechanisms that drive CRS [24]. Previous research supported findings related to the IL1B gene, which is vital in stimulating inflammation and cytokine cascades during CRS [25]. Likewise, earlier studies have identified IL15 as an essential modulator of T-cell growth and longevity, confirming its involvement in the hyperactive immune response characteristic of CRS [26]. The dysregulation of CD276, an immune checkpoint molecule, aligns with reports suggesting its role in immune evasion and enhanced inflammatory responses in the tumor microenvironment, which may intensify CRS during CAR T-cell therapy [27]. Additionally, NCR2, an activating receptor for NK cells, has been associated with heightened immune activation, further supporting its involvement in CRS pathology [28]. As reported, CCL17 is a chemokine that recruits T cells and DCs to sites of inflammation, and it may contribute to the immune cell infiltration and cytokine storm in CRS [29]. Together, our results are in agreement with existing literature, emphasizing the complicated nature of CRS and its reliance on these critical immune pathways.

Our results verified that the immune cell population, such as Mo, MFs, and DCs of hub genes, plays a major role in CRS pathogenesis. They also reveal involvement in the dysregulated immune responses and excessive cytokine production characteristic of CRS and the significant expression of key hub genes such as IL1B, IL15, CCL17, and CD276. A strategic approach to reduce CRS severity could involve focusing on these cell populations through altering their function or cytokine output.

One of the important therapeutic strategies specifically suppresses the activation of Mo or MFs, which helps to reduce the cytokine storm without negatively compromising the antitumor efficacy of CAR T-cells [23].

These hub genes are involved in essential pathways, such as cytokine-cytokine receptor interaction, IL17 signaling, and TNF signaling, which is further confirmed by enrichment analysis. These pathways were observed as the hyperinflammatory condition promoter in CRS. Moreover, this research identified potential therapeutic procedures, including medications such as canakinumab (targeting IL1B), ordesekimab (targeting IL15), and enoblituzumab (targeting CD276), which reduce CRS severity. However, the absence of drug interactions for NCR2 highlights gaps in existing treatment options, which require additional research into this gene and its associated pathways.

However, the limitations of the data regarding preinfusion samples restrict insights into postinfusion dynamics, which are crucial for understanding the full spectrum of CRS development. In addition, no drug interactions were identified for NCR2, highlighting a gap in therapeutic options. Key findings on hub genes and pathways lack experimental validation, requiring functional assays to confirm their roles in CRS; larger and postinfusion samples and prospective studies are required to confirm these results.

## Conclusions

The study highlights IL1B, IL15, CD276, NCR2, and CCL17 as key CRS genes in preinfusion CAR T-cell products. Their dysregulation activity may contribute to the increased inflammation noted in CRS, pointing to a loss of regulatory control. Bringing us closer to better patient outcomes, these findings not only suggest that these genes could serve as valuable biomarkers for predicting CRS but also open the way for the development of more precise treatments such as combining drugs such as enoblituzumab and canakinumab, which might assist in reducing CRS severity and making CAR T-cell therapy safer and more effective, ultimately improving patient lives.
